# Self‐Cooling Gallium‐Based Transformative Electronics with a Radiative Cooler for Reliable Stiffness Tuning in Outdoor Use

**DOI:** 10.1002/advs.202202549

**Published:** 2022-06-05

**Authors:** Sang‐Hyuk Byun, Joo Ho Yun, Se‐Yeon Heo, Chuanqian Shi, Gil Ju Lee, Karen‐Christian Agno, Kyung‐In Jang, Jianliang Xiao, Young Min Song, Jae‐Woong Jeong

**Affiliations:** ^1^ School of Electrical Engineering Korea Advanced Institute of Science and Technology (KAIST) Daejeon 34141 Republic of Korea; ^2^ School of Electrical Engineering and Computer Science Gwangju Institute of Science and Technology (GIST) Gwangju 61005 Republic of Korea; ^3^ Department of Mechanical Engineering University of Colorado Boulder CO 80309 USA; ^4^ Department of Electronics Engineering Pusan National University Busan 46241 Republic of Korea; ^5^ Department of Robotics Engineering Daegu Gyeongbuk Institute of Science and Technology (DGIST) Daegu 42988 Republic of Korea

**Keywords:** gallium, liquid metal, radiative cooling, stiffness tuning, transformative electronics

## Abstract

Reconfigurability of a device that allows tuning of its shape and stiffness is utilized for personal electronics to provide an optimal mechanical interface for an intended purpose. Recent approaches in developing such transformative electronic systems (TES) involved the use of gallium liquid metal, which can change its liquid–solid phase by temperature to facilitate stiffness control of the device. However, the current design cannot withstand excessive heat during outdoor applications, leading to undesired softening of the device when the rigid mode of operation is favored. Here, a gallium‐based TES integrated with a flexible and stretchable radiative cooler is presented, which offers zero‐power thermal management for reliable rigid mode operation in the hot outdoors. The radiative cooler can both effectively reflect the heat transfer from the sun and emit thermal energy. It, therefore, allows a TES‐in‐the‐air to maintain its temperature below the melting point of gallium (29.8 ℃) under hot weather with strong sun exposure, thus preventing unwanted softening of the device. Comprehensive studies on optical, thermal, and mechanical characteristics of radiative‐cooler‐integrated TES, along with a proof‐of‐concept demonstration in the hot outdoors verify the reliability of this design approach, suggesting the possibility of expanding the use of TES in various environments.

## Graphical abstract

Stiffness‐tuning reliability of gallium‐based transformative electronics in hot outdoor environment can be significantly enhanced by integrating a radiative cooler. It mitigates the influence of high outdoor temperature and sunlight exposure through solar reflection and thermal radiation, preventing unwanted softening of transformative electronics. Proof‐of‐concept demonstration of the radiative‐cooler‐integrated design suggests the expanded utility of transformative electronics with improved practicality toward outdoor use.

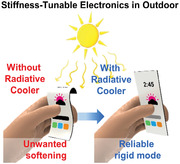

## 1 Introduction

**Figure 1:**
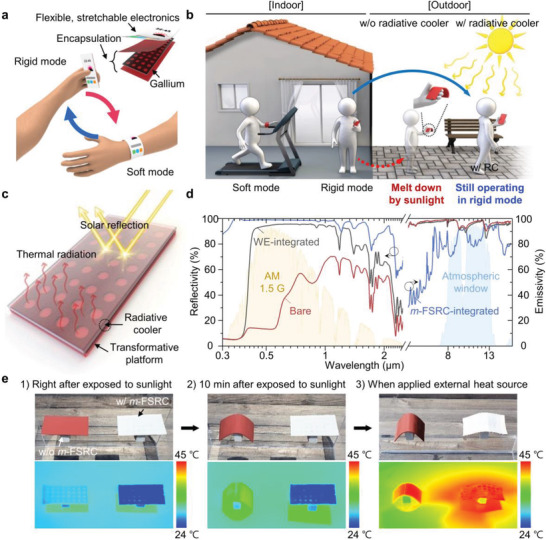
Overview of TES integrated with a multi‐layered, flexible, and stretchable radiative cooler (TES‐RC) that enables reliable rigid and soft operation modes in a hot outdoor environment. a) Schematic illustration of gallium‐based TES, which can convert between rigid handheld and soft wearable modes via temperature‐dependent stiffness tuning. The inset shows a basic structure of the TES, which is composed of a polymer‐encapsulated gallium frame and flexible, stretchable electronics. b) Conceptual diagram that compares rigid operation mode of TES without and with a radiative cooler in a hot environment under sunlight. TES‐RC allows maintenance of the rigid mode during outdoor use, whereas TES without a radiative cooler can soften due to temperature increase by sunlight. c) Schematic diagram illustrating the working principle of the radiative cooler on a transformative platform that reduces the overall device temperature through solar reflection and thermal radiation. d) Reflectivity and emissivity spectra from the visible to far‐infrared wavelength range for bare gallium‐based, white elastomer‐integrated, and *m*‐FSRC‐integrated transformative platforms. *m*‐FSRC indicates a multi‐layered, flexible, and stretchable radiative cooler. e) Optical and corresponding infrared (IR) images of transformative platforms with and without *m*‐FSRC integration.

Electronics are traditionally designed to have a fixed form factor that results in either a rigid or soft device structure. The distinctive mechanical nature of both rigid and soft electronics leads to unique properties of their own for specific applications. For instance, the robust nature and convenient handling of rigid electronics make them highly suitable for handheld or tabletop setups; the lack of surface conformability, however, limits their potential utilization for wearable applications. Soft electronics, on the other hand, are highly capable of dynamic shape deformation with potential utility for wearable applications, but they are inconvenient for off‐body handling due to their limited load‐bearing capacity.^[^ ^1^, ^2^, ^3^, ^4^, ^5^ ^]^ Although the conventional design structure of electronics is highly advantageous for specific applications, the finite mechanical properties of the current technologies restrict their potential use. One promising way to integrate the distinctive mechanical properties of both rigid and soft electronics is to merge flexible, stretchable electronics onto a transformative platform that can tune its rigidity as required. Recent advances in materials and device engineering have enabled such electronics, referred to as transformative electronic systems (TES),^[^ ^6^, ^7^, ^8^ ^]^ based on a thermally‐responsive platform built with a gallium‐elastomer composite (**Figure** **1**a). This emerging class of electronics can change their shape, stiffness, and stretchability through a temperature‐dependent liquid‐solid phase transition of the encapsulated gallium, thus enabling state conversion between soft and rigid modes. This allows the electronics to be used as not only rigid handheld electronics but also soft wearable devices to support desired applications.

While TES can provide stable and reliable mode conversion and operation in an environment where the ambient room temperature can be maintained below 30 ℃ (e.g., indoor environment; Figure 1b, left), controlling and keeping the desired operation mode in the outdoors is an arduous undertaking (Figure 1b, right). One of the most significant challenges in outdoor use of TES is preventing unwanted softening of a device when the rigid operation mode is desired in a hot environment (>30 ℃). During outdoor use, the TES inevitably accumulates undesired heat through sunlight absorption, ambient temperature transfer, and Joule heating of electronic components.

To enhance the practical utility of TES in real‐world applications, a technology that allows effective management of undesired excessive heat is needed. In the area of mobile and wearable electronics, various thermal management schemes have been actively investigated to prevent thermal discomfort to human skin as well as performance degradation of the device. For example, a thin metallic film^[^ ^9^ ^]^ and synthesized nanocomposites^[^ ^10^, ^11^, ^12^, ^13^ ^]^ have been studied as means to increase the thermal conductivity of electronics to effectively dissipate internally induced heat energy. However, this strategy cannot decrease the device temperature below its ambient temperature. This means that, when applied to TES in a hot environment with ambient temperature of over 30  ℃, this heat sink approach cannot cool the temperature of the device below 29.8 ℃, which is the melting point of gallium (i.e., the core material of TES). Integrating active cooling systems, such as coolant circulators^[^ ^14^, ^15^, ^16^ ^]^ or thermoelectric devices,^[^ ^7^, ^17^ ^]^ is an alternative option to address the thermal management issue, but it requires huge power consumption for long‐term device operation. Meanwhile, radiative coolers have been studied for on‐skin electronics to improve thermal comfort because they can provide heat dissipation without requiring external energy.^[^ ^18^, ^19^, ^20^, ^21^, ^22^, ^23^, ^24^ ^]^ To address the challenge associated with unwanted TES mode conversion in a hot outdoor environment, we investigated a radiative cooling system as a solution for thermal management of the TES. Here, we developed a multi‐layered, flexible, and stretchable radiative cooler (*m*‐FSRC) and integrated it with a TES to passively and effectively lower the device temperature through thermal radiation and solar reflection. This design allows the TES to maintain its rigid mode operation when favored, even under hot weather with strong sunlight exposure (Figure 1b, right). In addition, the highly flexible and stretchable properties of *m*‐FSRC preserve the natural deformation of the TES in soft mode operation. The following sections present the design and characterization of an *m*‐FSRC‐integrated TES and a proof‐of‐concept demonstration, verifying the potential of the proposed design to enable highly reliable “transformative” electronics that are not influenced by the environmental factor of ambient temperature.

## 2 Results and Discussion

### 2.1 Overview of TES‐RC Design

Figure 1c illustrates the concept of the TES integrated with *m*‐FSRC (TES‐RC), which lowers its temperature through radiative cooling. The radiative cooler not only releases internally induced heat energy, but also prevents energy absorption from the sun. These functions of radiative cooling eliminate constraints on the rigid mode operation of TES‐RC by decreasing the device temperature in outdoors, and the cooling effect is obviously strengthened due to the unique spectral properties of *m*‐FSRC. Figure 1d shows the reflectivity and emissivity of our *m*‐FSRC‐integrated transformative platform, in comparison to those of bare and commercial white elastomer (WE)‐integrated transformative platforms. The near‐unity reflectivity of *m*‐FSRC in the solar spectrum (i.e., 0.3–2.5 µm wavelength) effectively blocks energy gain from the sun, and the high emissivity in the atmospheric window (i.e., 8–13 µm wavelength) enables radiation of thermal energy from the device to outer space. Therefore, the TES‐RC can minimize the temperature increase during continuous operation in a hot outdoor environment with sunlight exposure.

The experiment shown in Figure 1e verifies the proposed design concept, highlighting the cooling effect of the *m*‐FSRC. The transformative platform integrated with the *m*‐FSRC retained its flat and stiff configuration when exposed to sunlight at ambient temperature of 32 ℃. On the contrary, a bare sample (i.e., no *m*‐FSRC integration) fully softened after 10 min exposure to sunlight. The corresponding IR images (Figure 1e, bottom) show the surface temperatures of the samples with and without the *m*‐FSRC (24 and 38 ℃, respectively), further highlighting its cooling performance for thermal management. The sample with the *m*‐FSRC was transformed to a soft state only when additional external heat (≈80 ℃) was applied. These results together indicate that the TES‐RC design can solve the problem associated with unwanted rigid‐to‐soft mode conversion of the TES in a hot outdoor environment, based on the outstanding cooling capability of the integrated *m*‐FSRC.

### 2.2 Optical/Thermal Characterization of *m*‐FSRC

**Figure 2:**
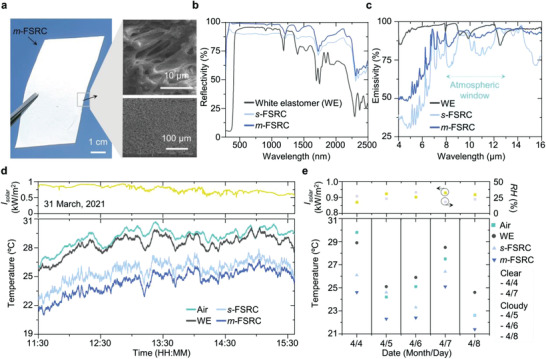
Optical characterization of *m*‐FSRC with thermal analysis. a) Photograph of *m*‐FSRC. The inset shows SEM images of *m*‐FSRC. b,c) Optical properties of WE, *s*‐FSRC, and *m*‐FSRC: b) reflectivity at the solar spectrum and c) emissivity at the atmospheric window. d,e) Temperature changes of ambient air, WE, *s*‐FSRC, and *m*‐FSRC d) during the daytime and e) over several days, highlighting the superior cooling effect of *m*‐FSRC to air, WE, and *s*‐FSRC. *I_solar_* and *RH* indicate average solar intensity and relative humidity, respectively.

**Figure** **2**a displays a photograph and a scanning electron microscope (SEM) image of the *m*‐FSRC. The *m*‐FSRC consists of five single‐layered FSRCs (*s*‐FSRCs) with porous structures (Figure S1, Supporting Information). The micro‐scale pores lead to multiple Mie scattering,^[^ ^25^ ^]^ hence remarkably enhancing the reflectivity within the solar spectrum (i.e., 0.3–2.5 µm wavelength). Figure 2b,c shows the spectral characteristics of the WE, *s*‐FSRC, and *m*‐FSRC. Figure 2b spectrally demonstrates that samples with a porous structure (i.e., *s*‐FSRC and *m*‐FSRC) present higher reflectance than the WE in the solar spectrum, especially in the near‐infrared spectral range (i.e., 1.3–2.5 µm wavelength). Since the energy absorption in the near‐infrared range accounts for a considerable amount (11.6%) of total solar energy, a strong reflection of FSRCs in the near‐infrared regime is highly advantageous for radiative cooler design.^[^ ^26^ ^]^ The *s*‐FSRC (140 µm in thickness) shows ≈90% solar reflectivity and the *m*‐FSRC (700 µm in thickness) achieves near‐unity reflectivity. Increasing the thickness of the *s*‐FSRC can also be an option to enhance solar reflectivity because it increases the probability of incident photons undergoing multiple Mie scattering. However, the number of pores saturates during a drying process when thickening the *s*‐FSRC, resulting in a steep decrease in the solar reflectivity (Figure S2, Supporting Information). For this reason, instead of thickening the *s*‐FSRC, we stacked multiple layers of *s*‐FSRCs to create numerous pores for enhanced solar reflectivity through the thermal bonding process enabling a robust adhesion (Figure S3, Supporting Information). The detailed fabrication process of the *m*‐FSRC is presented in the Experimental Section. Furthermore, multiple layers of the porous structure of the *m*‐FSRC (five layers in this work) enhance emissivity in the atmospheric window, compared to the WE and *s*‐FSRC (Figure 2c).

Based on its outstanding optical characteristics, the *m*‐FSRC is expected to achieve exceptional cooling performance. To demonstrate sub‐ambient cooling by the fabricated *m*‐FSRC samples, thermocouples were attached to the backside of the samples to record the temperature changes and an ambient air box was installed to prevent self‐heating of the ambient air sensor (Figure S4, Supporting Information). In this measurement setup, the *m*‐FSRC shows sub‐ambient cooling of ≈4.5 ℃ under peak solar power of 927 W m^−2^, while the WE and *s*‐FSRC cool the surface temperature by only about 1 and 3.5 ℃, respectively (Figure 2d). The cooling power and cooling temperature were calculated to prove the performance of the *m*‐FSRC in various ambient temperatures (from 0 to 50 ℃; Figure S5, Supporting Information). In addition, the daily measurement over 5 days further confirms that the *m*‐FSRC has the highest cooling performance among the comparison group regardless of the weather conditions (Figure 2e and Figure S6 in Supporting Information).

### 2.3 Thermo‐Mechanical Studies of TES‐RC

**Figure 3:**
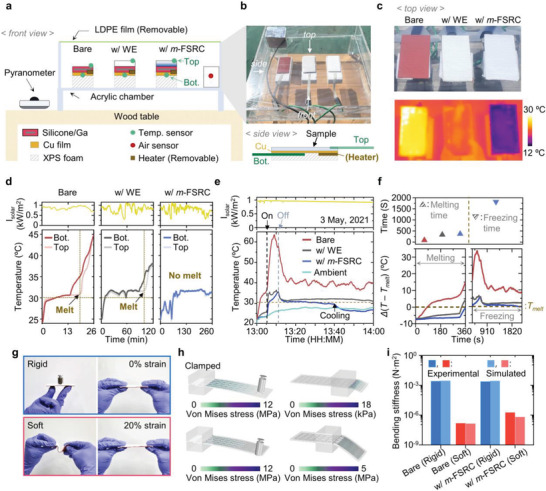
Thermo‐mechanical studies of the gallium‐based transformative platform with the integration of *m*‐FSRC. a) Schematic diagram and b) corresponding photograph showing the setup for testing the temperature response of transformative devices with no attachment and with WE and *m*‐FSRC integration in the controlled outdoor environment. Each sample was placed in the acrylic chamber and the chamber was covered by LDPE film to prevent convection heat flow. The inset in b) shows the side view of the test setup with a heater and temperature sensors. c) Photograph (top) of the experimented samples (i.e., Bare, w/ WE, and w/*m*‐FSRC) with corresponding IR images (bottom), comparing their cooling capability. d,e) Temperature measurement of the samples in the test configuration during d) thawing (heat source: the sun) and e) cooling following thawing by heater. f) Magnified view of Figure 3e showing the transition section of each of the melting and freezing processes. g) Optical images of the transformative platform with *m*‐FSRC in rigid (top) and soft (bottom) modes. h) Finite element analysis (FEA) simulation results for bending stiffness of the transformative platform without and with the integration of *m*‐FSRC. i) Experimental and FEA simulation result plots corresponding to Figure 3h.

After verifying the cooling performance of the *m*‐FSRC, the radiative cooler was integrated on a transformative platform. For thermal characterization of the transformative device, the measurement setup was installed as shown in **Figure** **3**a,b. An acrylic chamber was placed on a wooden table with a height of ∼1 m to avoid heat influence from the ground, and temperature sensors were attached on both the top and the bottom sides of the various samples: 1) a bare gallium‐based transformative platform without a radiative cooler (Bare); 2) a WE‐integrated transformative platform (w/ WE); and 3) a *m*‐FSRC‐integrated transformative platform (w/*m*‐FSRC). Here, extruded polystyrene (XPS) foam boxes beneath the samples provide thermal isolation from the bottom surface of the acrylic chamber and the heaters (Figure 3b, bottom) enable temperature control of the samples by heat conduction through the copper (Cu) layer (Figure 3b). Finally, a convection shield (i.e., low‐density polyethylene; LDPE film) covered the measurement setup to produce a harsh environment with high air temperature by reducing the convective heat exchange.

Figure 3c shows thermographic images of the test samples captured by an IR camera in this experimental setup. Since all the samples have similar thermal emissivity, the color in IR images indicates the surface temperature of the samples. In this context, the w/*m*‐FSRC shows remarkably low temperature compared to the other samples (Figure 3c, bottom). Figure 3d demonstrates that the *m*‐FSRC can provide thermal protection from strong sunlight exposure. During the measurement, the temperature differences between the top and the bottom surfaces were observed in all the samples. The heat capacity of the gallium‐based transformative platform can induce a temperature difference between the top and bottom surfaces unless the device effectively cools down. According to our experiment (Figure 3d), the Bare and w/ WE samples show a relatively large temperature difference between the top and the bottom, whereas the w/*m*‐FSRC sample exhibits a negligible temperature gap, indicating its excellent cooling capability. Because the bottom surfaces face the XPS foam and the top surfaces are open, the heat is easily accumulated at the bottom. Thus, if the cooling of the device structure is not sufficient, gallium at the bottom side will melt first and temperature lags between the top and the bottom will appear, as shown in the Bare and w/ WE samples. Considering the temperature changes of the top surfaces, the Bare and w/ WE completely melt by solar energy absorption in 16 and 99 min, respectively, and the temperature difference between the top and the bottom thereafter disappeared. On the contrary, the w/*m*‐FSRC sample showing no temperature difference successfully prevents the liquefaction of gallium by protecting it from strong sunlight.

Figure 3e exhibits the self‐cooling feature of the sample with the *m*‐FSRC. First, the samples are placed on the top of copper plates to transfer the heat uniformly, and they are thawed by the heaters (operation power = 610.3 W m^−2^; Figure S7, Supporting Information), which are placed at the bottom side of the copper plates. After gallium in the samples is fully melted, the heaters are turned off. The Bare and w/ WE samples maintain their soft states at temperatures above the melting point of gallium (Figure 3e; red and black lines). In contrast, the self‐cooling ability of the w/*m*‐FSRC sample enables its temperature to be decreased below the melting point of gallium (Figure 3e; blue line). Figure 3f shows an enlarged view of time intervals during the melting and freezing processes in Figure 3e. The *m*‐FSRC not only enables the longest duration of solid‐state prior to melting (Figure 3f, left) but also facilitates the liquid‐to‐solid conversion of gallium through effective self‐cooling (Figure 3f, right). These results together show that TES‐RC can maintain the rigid operation mode under extremely hot weather through significant cooling enabled by both effective thermal radiation and solar reflection (Figure S8, Supporting Information). In addition, the w/*m*‐FSRC sample maintains the soft state when 33 ℃ heat is applied, which means 33–35 ℃ skin temperature allows the TES‐RC to be used as a wearable form in an outdoor environment (Figure S9a, Supporting Information). The release from the skin can then make the w/*m*‐FSRC sample convert into rigid mode while the Bare sample cannot implement the soft‐to‐rigid mode conversion due to its insufficient cooling ability (Figure S9b, Supporting Information).

These thermal characteristics of the w/*m*‐FSRC device significantly enhance the reliability of the TES mode conversion (between the rigid and soft state), helping provide the desired mechanical properties for targeted use. Figure 3g shows the distinct mechanical properties of the w/*m*‐FSRC device, which can bear a high load and maintain its flat configuration in the rigid mode and can be twisted and stretched in the soft mode. The experimental and simulation results (Figure 3h) reveal that the device can tune its bending stiffness substantially (over three orders of magnitude) through mode switching. In addition, we observed that integration of the *m*‐FSRC onto a transformative platform has a minor effect on stiffness in soft mode due to the highly flexible and stretchable nature of the *m*‐FSRC, as demonstrated in Figure 3i.

### 2.4 Application Demonstration of TES‐RC

**Figure 4:**
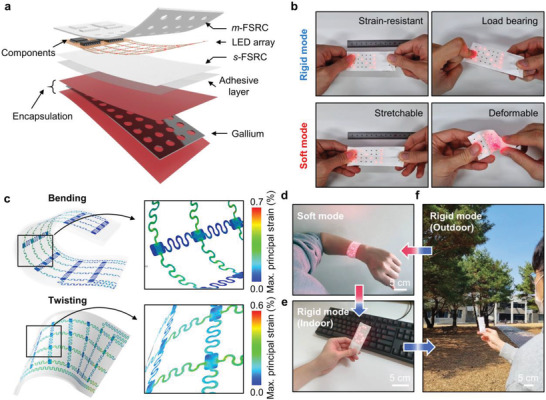
Application demonstration of TES‐RC that can convert between soft wearable and rigid handheld electronics. a) Exploded‐view schematic diagram that illustrates TES‐RC consisting of a stretchable LED array, a radiative cooler and a transformative platform. b) Optical images of the device comparing mechanical properties in rigid (top) and soft (bottom) modes. c) FEA results for the device in a soft state under bending and twisting. d‐f) Photographs of the TES‐RC demonstrating reliable rigid‐soft mode conversion and stable operation in d) soft wearable form as well as in rigid handheld setup during both e) indoor and f) outdoor use.

For a proof‐of‐concept demonstration, we manufactured a multi‐purpose optoelectronic device with a TES‐RC design, which can be used in both handheld and wearable configurations. **Figure** **4**a shows an exploded view of our demonstrative device, consisting of a transformative platform with a gallium frame, a stretchable light‐emitting diode (LED) array, and the *m*‐FSRC. The detailed process to fabricate the device is described in the Experimental Section. Note that polydimethylsiloxane (PDMS) is used as the adhesive layer to bond the *m*‐FSRC‐encapsulated LED array with the transformative platform. The LED array is integrated on a stretchable printed circuit board (PCB) with filamentary serpentine mesh interconnects (Figure S10, Supporting Information). This mesh structure functions as a window to maximize the cooling effect of the *m*‐FSRC. In addition, the serpentine interconnects enable stretching of the device in soft mode. To allow light emission from LEDs, through‐holes are created in the *m*‐FSRC in accordance with the position of the LEDs. The encapsulation with the punched *m*‐FSRC minimizes heat absorption by the LED array circuit, leading to cooling performance equal to that of the fully‐covered w/*m*‐FSRC sample (Figure S11, Supporting Information). Meanwhile, other designs with the LED array placed on the top layer show poor cooling performance due to the adverse effect of the exposed LED array circuits.

Figure 4b presents the representative behaviors of the transformative optoelectronic device when an external force is applied. In rigid mode, the device exhibits high resistance against deformations such as strain and bending. On the contrary, the soft‐mode device becomes stretchable and deformable due to the liquefaction of the gallium frame. The FEA further verifies that the device in the soft form can accommodate large deformation (bending, twisting, and 20% uniaxial stretching) due to the low‐modulus, the elastic nature of the *m*‐FSRC and the LED array circuit with a filamentary serpentine design (Figure 4c and Figure S12 in Supporting Information).

Empowered by the cooling ability of the integrated *m*‐FSRC, the transformative optoelectronic device offers the desired operation mode (either rigid or soft mode) in both indoor and outdoor environments, especially by minimizing the unwanted softening of the device in a hot outdoor environment (Figure 4d–f). In soft mode, the device could be used as a wearable display, conformably wrapping on the wrist, as shown in Figure 4d. After being converted to rigid mode, the device could be transformed into a handheld display and remain flat and stiff in an indoor environment where the room temperature was controlled well below 30 ℃ (i.e., melting temperature of gallium; Figure 4e). When the user moves to a hot outdoor environment (ambient temperature of 32 ℃) with strong sunlight, the device could still maintain its rigid mode due to the cooling effect provided by the *m*‐FSRC, verifying the concept of the TES‐RC design (Figure 4f).

## 3 Conclusion

In summary, the TES‐RC design enables highly reliable transformative electronics that mitigate the influence of high outdoor temperature and sunlight exposure. The integrated *m*‐FSRC effectively reflects solar energy and radiates thermal energy, thus cooling the TES to prevent unwanted mode conversion from rigid to the soft mode in a hot outdoor environment. The effective cooling ability, zero power requirement, and the stretchability of the *m*‐FSRC make it a highly favorable option for integration with TES in terms of thermal management and mechanical tuning of the device structure. Exploring this option in connection with flexible thermoelectric devices^[^ ^7^ ^]^ for hybrid operation is a promising direction for future TES that enable both fast stiffness tuning (by active temperature control) and energy‐free, environment‐independent maintenance of the desired TES operation mode (by passive radiative cooling).

## 4 Experimental Section

### Fabrication of *m*‐FSRC

Styrene‐ethylene‐butylene‐styrene (SEBS) beads (Tuftec^TM^ H1062, Asahi Kasei), isopropyl alcohol (IPA), and chloroform (C2432, Sigma‐Aldrich) were mixed in a mass ratio of 3.5:81:15.5 at room temperature. The mixture was sonicated for 1 day to dissolve SEBS beads. The SEBS solution was then poured on a glass slide to evaporate chloroform and IPA (Figure S13a, Supporting Information). After evaporation for 1 day, the SEBS was chained with air voids to form the *s*‐FSRC. After repetitive fabrication of the *s*‐FSRC, five different *s*‐FSRC layers were peeled from the glasse slides and stacked for thermal bonding at 70 °C for 60 min with minimized pressure to create the *m*‐FSRC. Note that five *s*‐FSRCs are optimized to create *m*‐FSRC for both high cooling performance and flexibility (Figure S14, Supporting Information) and excessive pressure during thermal bonding can damage the pores, degrading the solar reflectivity of *m*‐FSRC (Figure S15, Supporting Information).

### Fabrication of a Gallium‐Based Transformative Platform

A gallium frame was first fabricated by the freeze‐casting method. Liquid gallium (Ga metal 99.99, Rotometals) was cast on the PDMS mold and clamped tightly with a glass substrate. The gallium‐filled mold was placed on a cool plate (CP‐200TT, TE Technology) with the temperature set to 5 ℃ to freeze the liquid gallium. The solidified gallium frame was released from the mold and then encapsulated with silicone elastomer (RT623 A/B, mixing ratio 9:1) to create a transformative platform.

### Fabrication of the Optoelectronic Device with TES‐RC Design

The optoelectronic device with the TES‐RC design consisted of a transformative platform, a stretchable LED array, and the *m*‐FSRC. The transformative platform and the *m*‐FSRC were prepared as described in the previous sections. Note that the *m*‐FSRC was additionally patterned by laser cutting to create holes for LED light emission (Figure S13b, Supporting Information). To build a stretchable LED array, a stretchable PCB was fabricated on a Kapton film (120 µm in thickness) with copper interconnects (18 µm in thickness). A microcontroller unit (ATMEGA328P‐AU, Microchip Technology, Inc.), two 8‐bit shift registers (74HC595D, Toshiba), a slide switch (JS102011JCQN, C&K), and LEDs (APG0603SEC‐E‐TT, Kingbright) were then soldered on the PCB using a low‐temperature soldering paste (SMDLTMFP10T5, ChipQuik) to create a stretchable LED array. For the integration of the whole system, the LED array was first encapsulated with the *m*‐FSRC. A single *s*‐FSRC layer was placed beneath the LED array to make thermal bonding (at 70 ℃ for 60 min) with the patterned *m*‐FSRC on top of the LED array. Subsequently, the FSRC‐encapsulated LED array was integrated on top of the transformative platform. For the bonding, a PDMS layer (10:1 ratio of base elastomer and curing agent; 100 µm in thickness) was spin‐coated on the transformative platform and partially cured at 70 ℃ for 20 min. The encapsulated LED array was then placed and thermal treatment was carried out at 70 ℃ for 60 min to complete the device fabrication.

### Structural and Spectral Analysis

The optical reflectance of the *s*‐FSRC, WE, and *m*‐FSRC in a wavelength range of 280  to 2500 nm was measured using an ultraviolet‐visible‐near‐infrared spectrophotometer (Lambda 950, Perkin Elmer, Inc.) with an integrating sphere. In addition, a Fourier transform infrared spectrometer (VERTEX 70v, Bruker) with an Au‐coated integrating sphere was used to derive the emissivity spectra (i.e., *E* = 1 – *R* + *T* where *E*, *R*, and *T* denote emissivity, reflectivity, and transmittance, respectively). Structural analyses were conducted by using a SEM (S‐4700, Hitachi Hi‐Tech).

### Thermal Characterization of Transformative Platforms with Radiative Coolers

The experimental setup for thermal characterization of the transformative devices with radiative coolers is illustrated in Figures 3a,b. For reliable thermal characterization to investigate the cooling performance of the devices, an XPS foam was prepared to provide thermal insulation between the transformative devices and the bottom surface of the acrylic chamber. The double‐sided thermal conductive tape was then laminated on the thermal insulation blocks, and adhesive temperature sensors (ST‐50, RKC Instrument Inc.) were placed on the top and the bottom sides of the samples. An ambient air sensor was installed in an Al‐coated paper box to measure the temperature of the naturally convective air in the chamber. This box prevents the ambient air sensor from overheating by solar irradiance. Finally, the whole acrylic chamber was covered by an LDPE film to suppress the convection effect, and a pyranometer (CMP6, Kipp & Zonen) was placed beside the acrylic chamber to measure solar irradiance.

### Measurement of Bending Stiffness

To measure the bending stiffness of the transformative platform, one end of the device sample was clamped, and deflection of the free end was measured. The deflection of the free end was induced by its own weight and a 20‐g weight for soft‐ and rigid‐mode platforms, respectively. The bending stiffness was then derived from the measured deflection results and the equation *E̅I̅ = FL^3^*/3z, where *F*, *z*, and *L* indicate the applied force, deflection of the free end, and length of the beam, respectively.

### Mechanical Modeling and Analysis

For the mechanical simulations, commercial FEA software (ABAQUS, Dassault Systèmes) was used. Each component such as the gallium frame, silicone encapsulation (RT623, ELASTOSIL), *m*‐FSRC, and stretchable PCB was constructed by a 3D rendering program. The stretchable PCB and the other components were simulated using four‐node shell elements (S4R) and eight‐node, 3D hexahedron elements (C3D8R), respectively. The results provided information on the bending stiffness and mechanical stresses of transformative electronics under various deformation conditions. The mechanical properties of the components used in the simulation can be found in Table S1 (Supporting Information).

### Experiments on Human Subjects

All experiments on human skins were performed under approval from the Institutional Review Board at Korea Advanced Institute of Science and Technology (protocol number: KH2018‐35) and received informed consent from the volunteer subjects.

## Conflict of Interest

The authors declare no conflict of interest.

## Supporting information

Supporting Information

## Data Availability

The data that support the findings of this study are available from the corresponding author upon reasonable request.
