# Supplementary material for: Self‐Cooling Gallium‐Based Transformative Electronics with a Radiative Cooler for Reliable Stiffness Tuning in Outdoor Use
Source: Adv Sci (Weinh). 2022 Jun 5;9(24):2202549. doi: 10.1002/advs.202202549 (PMC9404411; doi:10.1002/advs.202202549)
Supplement: Supplementary file 1 — Supporting Information [file ADVS-9-2202549-s001.pdf]

ADVANCED SCIENCE
Open Access

Supporting Information

for *Adv. Sci.*, DOI 10.1002/advs.202202549

Self-Cooling Gallium-Based Transformative Electronics with a Radiative Cooler for Reliable Stiffness Tuning in Outdoor Use

Sang-Hyuk Byun, Joo Ho Yun, Se-Yeon Heo, Chuanqian Shi, Gil Ju Lee, Karen-Christian Agno, Kyung-In Jang, Jianliang Xiao, Young Min Song and Jae-Woong Jeong**

Supporting Information

Self-Cooling Gallium-Based Transformative Electronics with a Radiative Cooler for Reliable Stiffness Tuning in Outdoor Use

Sang-Hyuk Byun[†]*, Joo Ho Yun*[†]*, Se-Yeon Heo, Chuanqian Shi, Gil Ju Lee, Karen-Christian Agno, Kyung-In Jang, Jianliang Xiao, Young Min Song*, and Jae-Woong Jeong**

Component	Material	Young's modulus	Density	Poisson's ratio
**Gallium frame**	Gallium (Solid)	9.8 GPa	5910 kg/m^3	0.465
	Gallium (Liquid)	~0 Pa	6095 kg/m^3	~0.5
**Polymer encapsulation**	RT623	290 kPa	1120 kg/m^3	~0.5
**Radiative cooler**	*p*-SEBS	200 kPa	250 kg/m^3	~0.5
**PCB**	Polyimide	3.1 GPa	1300 kg/m^3	0.34
	Copper	120 GPa	8960 kg/m^3	0.34

Table S1. Mechanical properties of the components used in this study to fabricate the radiative-cooler-integrated transformative electronics.

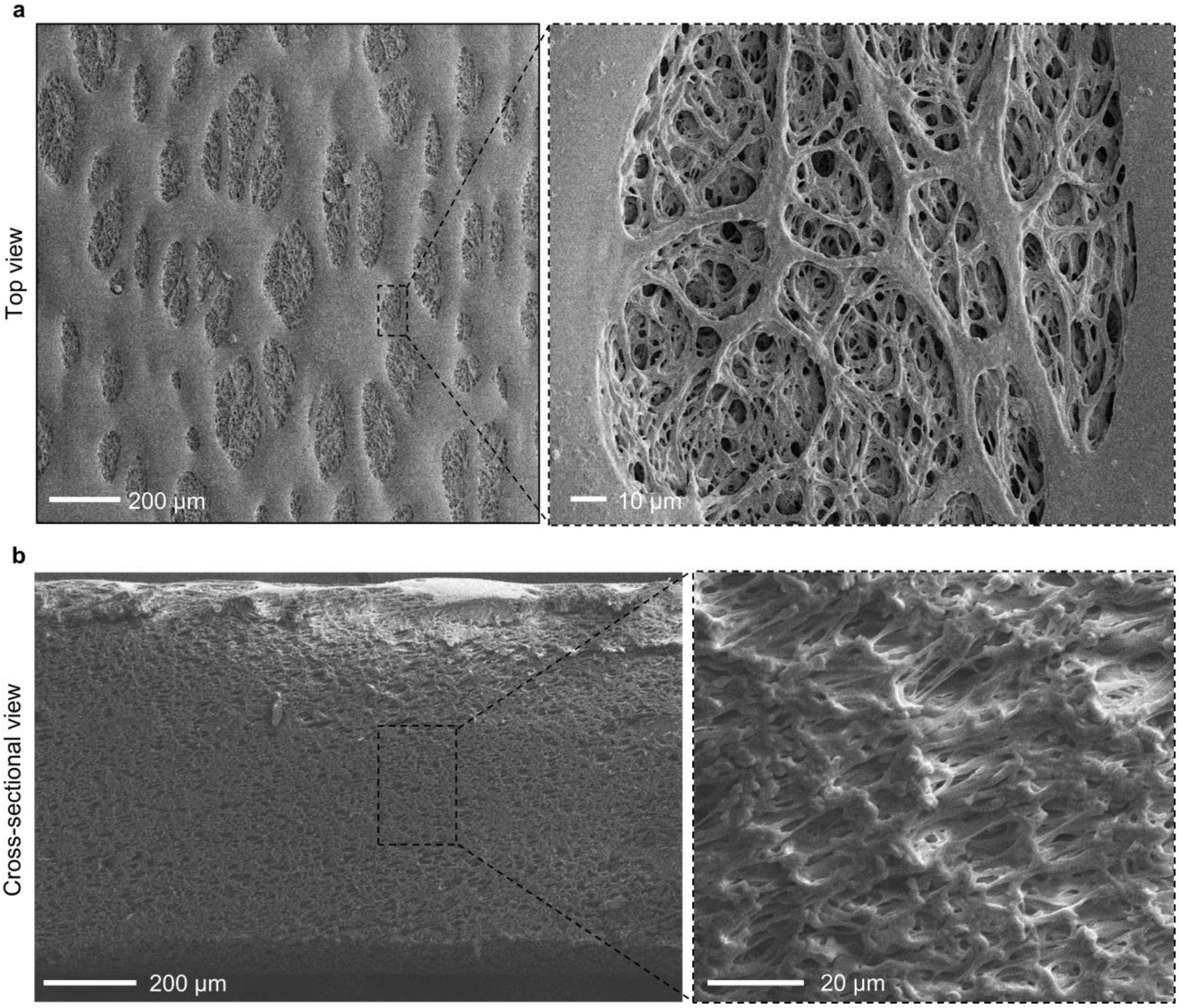

Figure S1. Scanning electron microscope images of *m*-FSRC with porous structures. a) Top view of *m*-FSRC. b) Cross-sectional view of *m*-FSRC which consists of 5 layers of *p*-SEBS with the thickness of 700 μm. The stacked structure allows to have numerous pores which can effectively reflect the solar energy by Mie scattering.

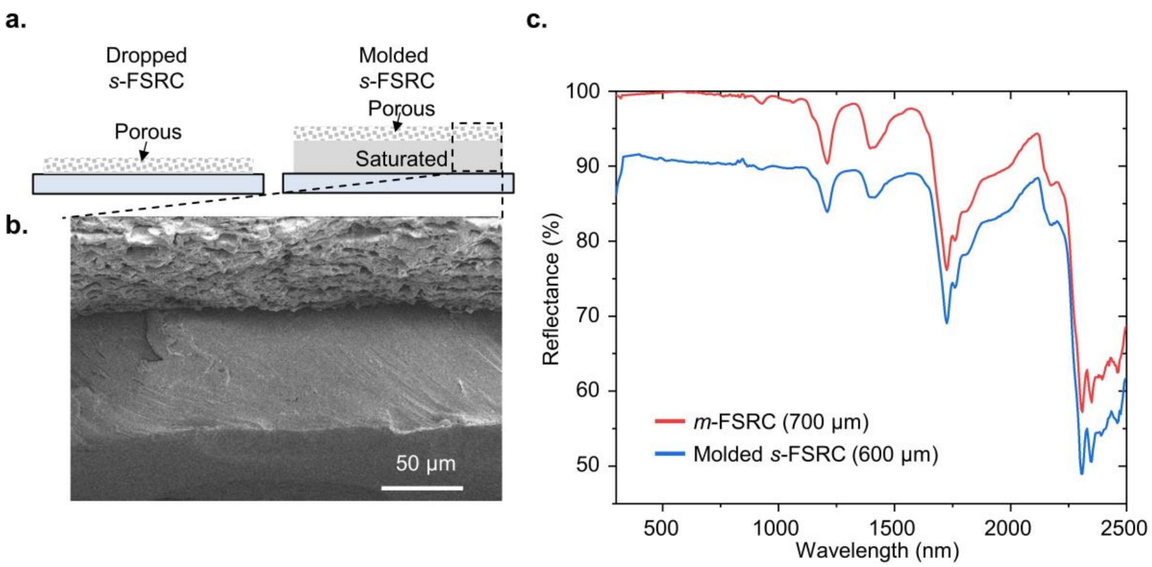

Figure S2. Comparison of *s*-FSRCs fabricated by drop casting and molding. (a) Schematic diagram illustrating the vaporization process of the drop-casted *s*-FSRC (140 μm in thickness) and the molded *s*-FSRC (600 μm in thickness). (b) SEM image of the molded *s*-FSRC, showing the saturated region at the bottom surface without pores (c) Solar reflectance of *m*-FSRC and the molded *s*-FSRC.

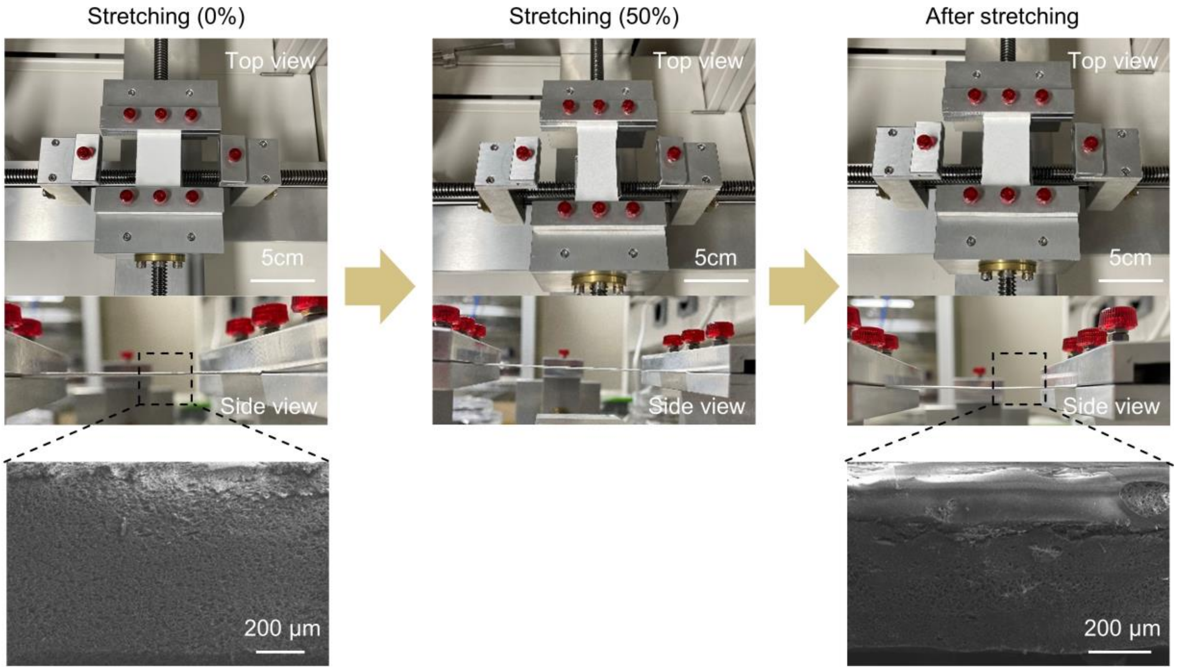

Figure S3. The stretchability test of *m*-FSRC with 50% strain to show the robust interface of thermally bonded *s*-FSRCs

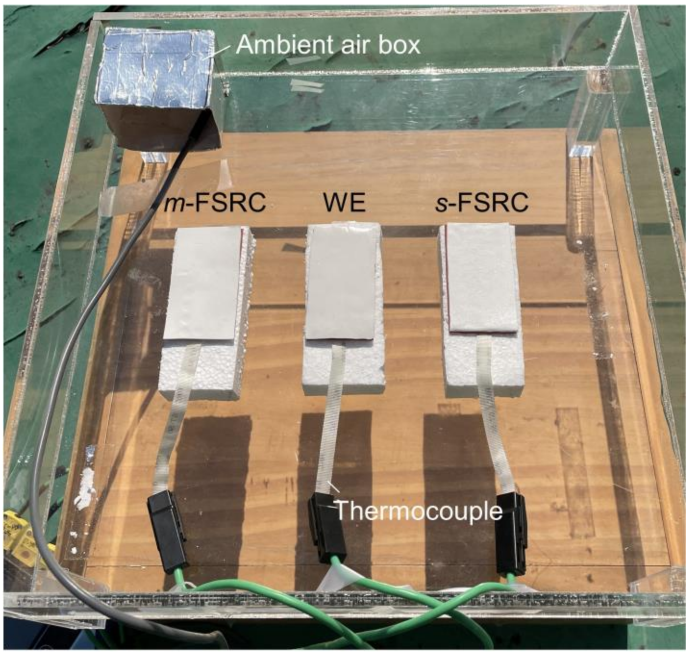

Figure S4. Measurement setup to characterize cooling performance of WE, *s*-FSRC, and *m*-FSRC. Thermocouples were attached to the backside of the samples to record the temperature changes and an ambient air box was installed to prevent self-heating of ambient air sensor.

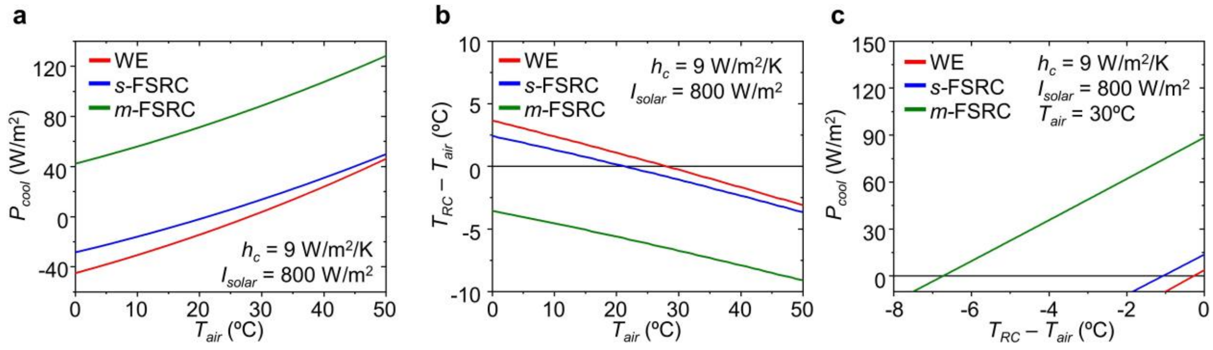

Figure S5. Theoretical cooling performance of WE, *s*-FSRC, and *m*-FSRC. a,b) Calculated results of a) cooling power (P_{cool}) and b) cooling temperature ($T_{RC} - T_{air}$) using thermal equilibrium equation with non-radiative heat exchange coefficient h_c = 9 W/m^{2}K and solar intensity I_{solar} = 800 W/m^2. T_{RC} and T_{air} indicate temperature of the integrated radiative cooler and ambient air, respectively. c) Cooling power as a function of cooling temperature at 30 ℃ of ambient temperature.

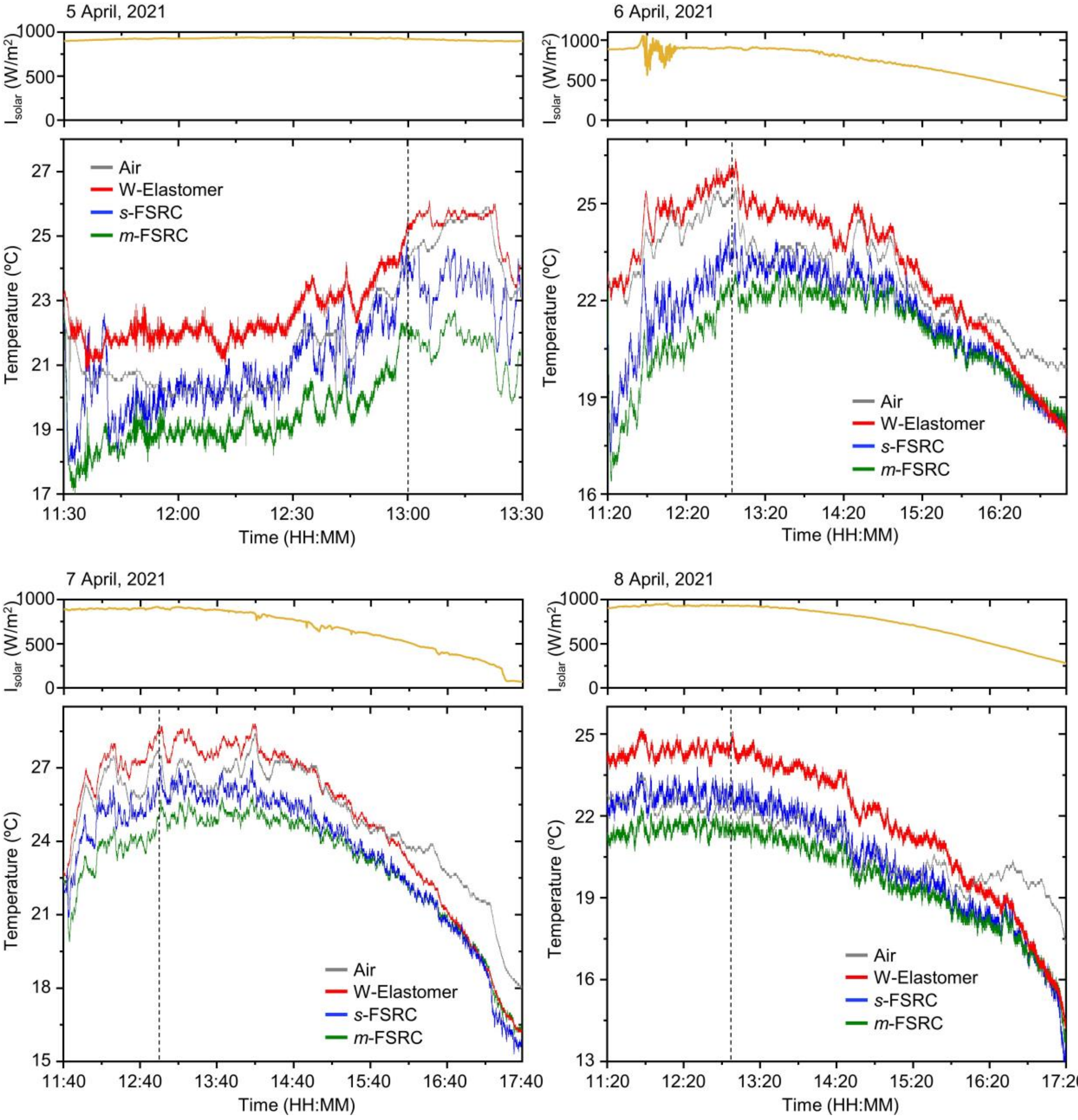

Figure S6. Temperature changes of ambient air, WE, s-FSRC, and *m*-FSRC over several days in the daily measurement.

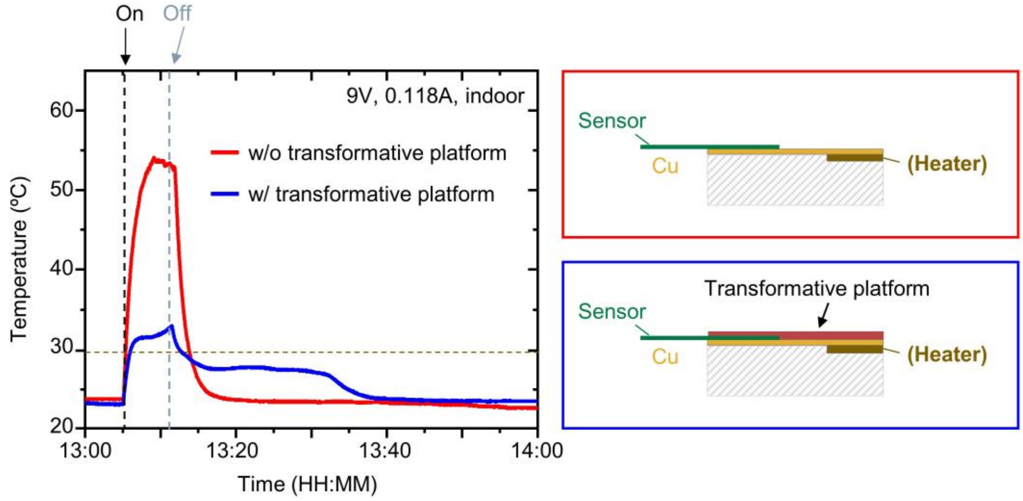

Figure S7. Temperature changes of heaters in an indoor environment with operation power of 610.3 W/m^2. The right image shows the test setup for the measurement.

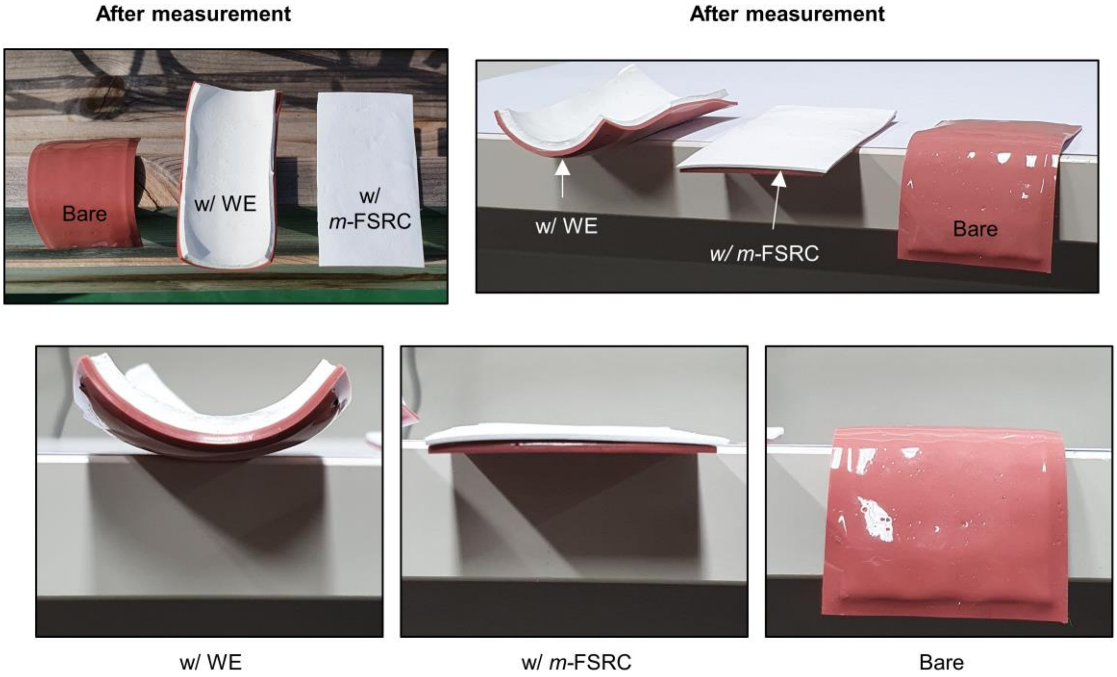

Figure S8. Photographs of the test samples (Bare, w/ WE, and w/ *m*-FSRC), showing the geometrical changes after exposed to strong sunlight. The w/ *m*-FSRC sample maintains its rigid mode without shape deformations.

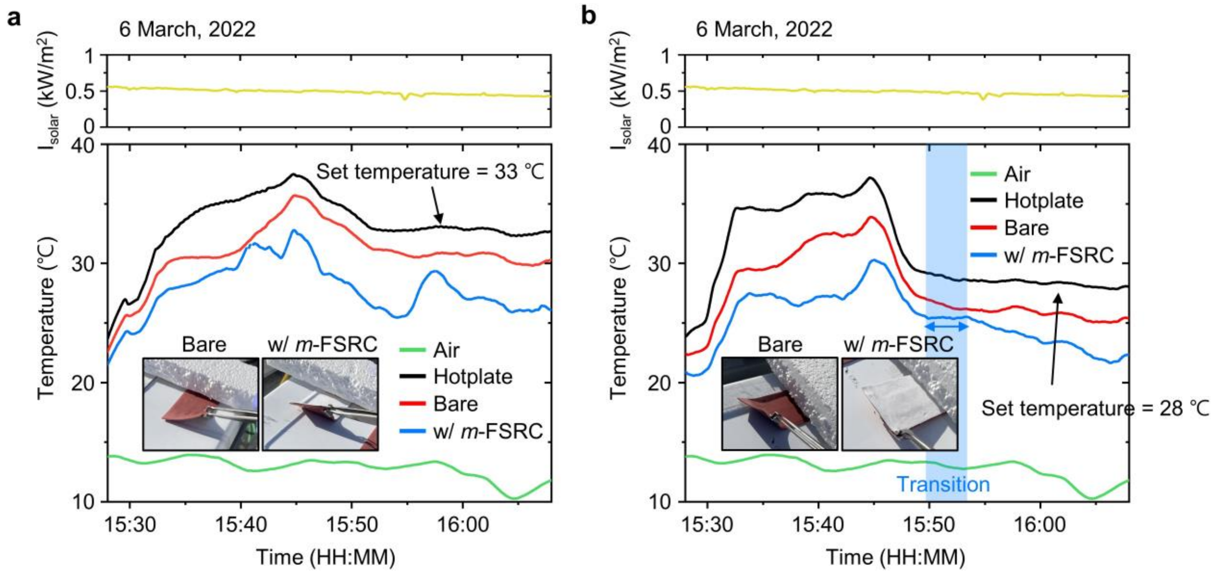

Figure S9. Thermal behavior of Bare and w/ *m*-FSRC samples when placed on the hotplate with the temperature of a) 33 ℃ and b) 28 ℃. The samples were first fully melted at 35 ℃. The inset shows mechanical properties of the samples at the end of measurement.

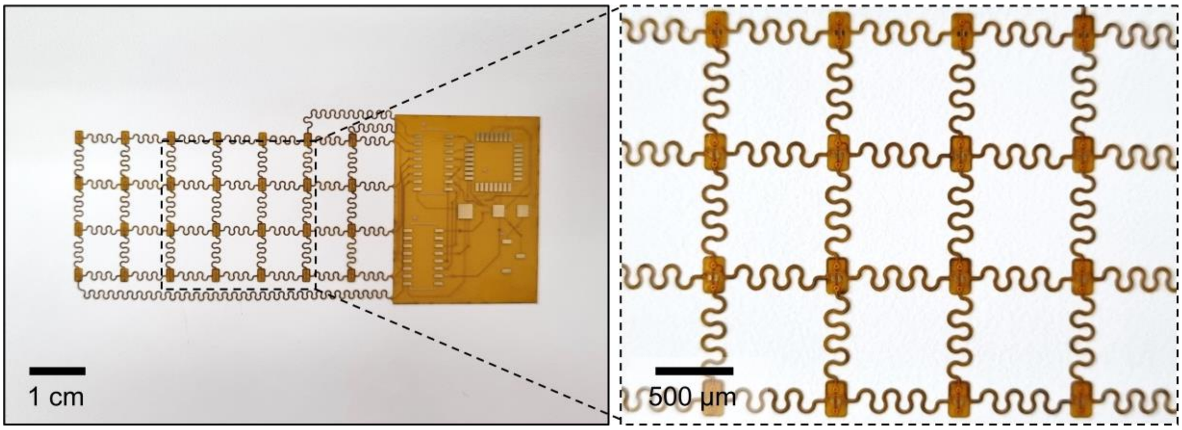

Figure S10. Optical image of a stretchable PCB with filamentary serpentine mesh interconnects for LED array operation.

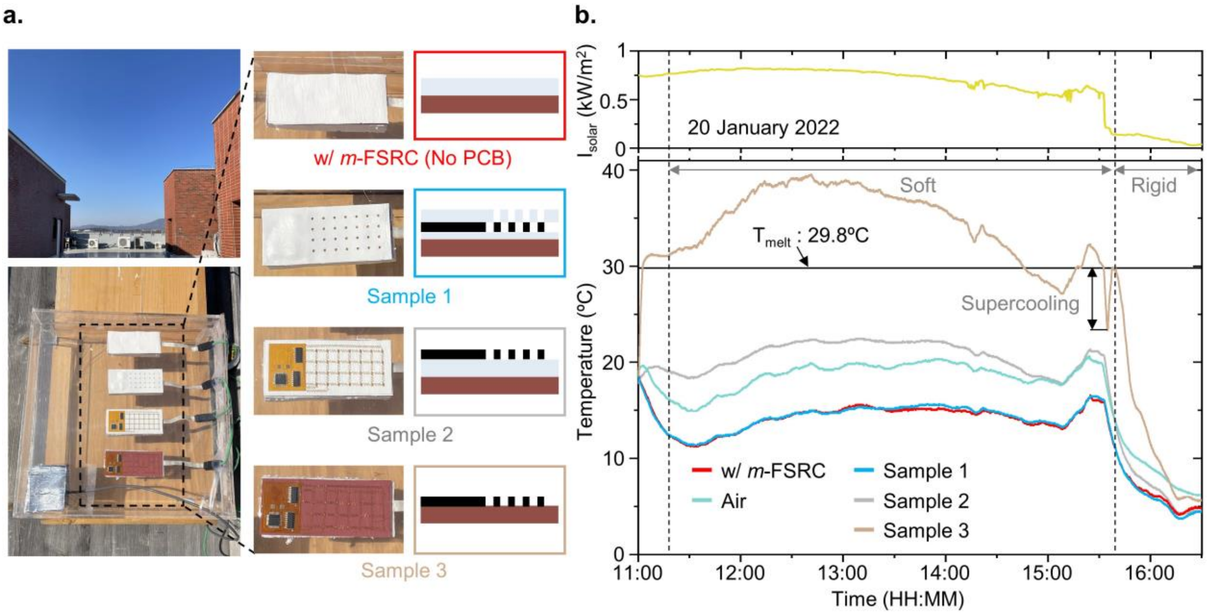

Figure S11. Comparison of cooling performance between various integration methods to fabricate transformative optoelectronic device. a) Photographs of the test setup and samples made with different integration approaches. A bare transformative platform with *m*-FSRC (w/ *m*-FSRC) is used as a control to evaluate cooling performance of the three different samples. Sample 1 has *m*-FSRC at the top layer to cover the entire LED circuit except the LEDs for LED light emission. Meanwhile, in the two other samples, the LED array circuit is integrated either on top of w/ *m*-FSRC (Sample 2) or bare transformative platform (Sample 3). b) Temperature changes of the test samples during the daytime measurement, showing superior cooling performance of Sample 1 to the ones of the other two samples.

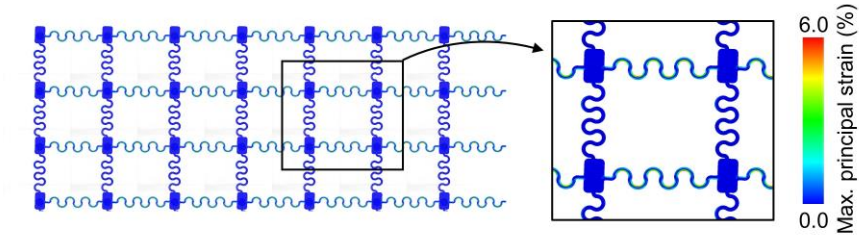

Figure S12. FEA results for the optoelectronic device in a soft state under uniaxial stretching (20%).

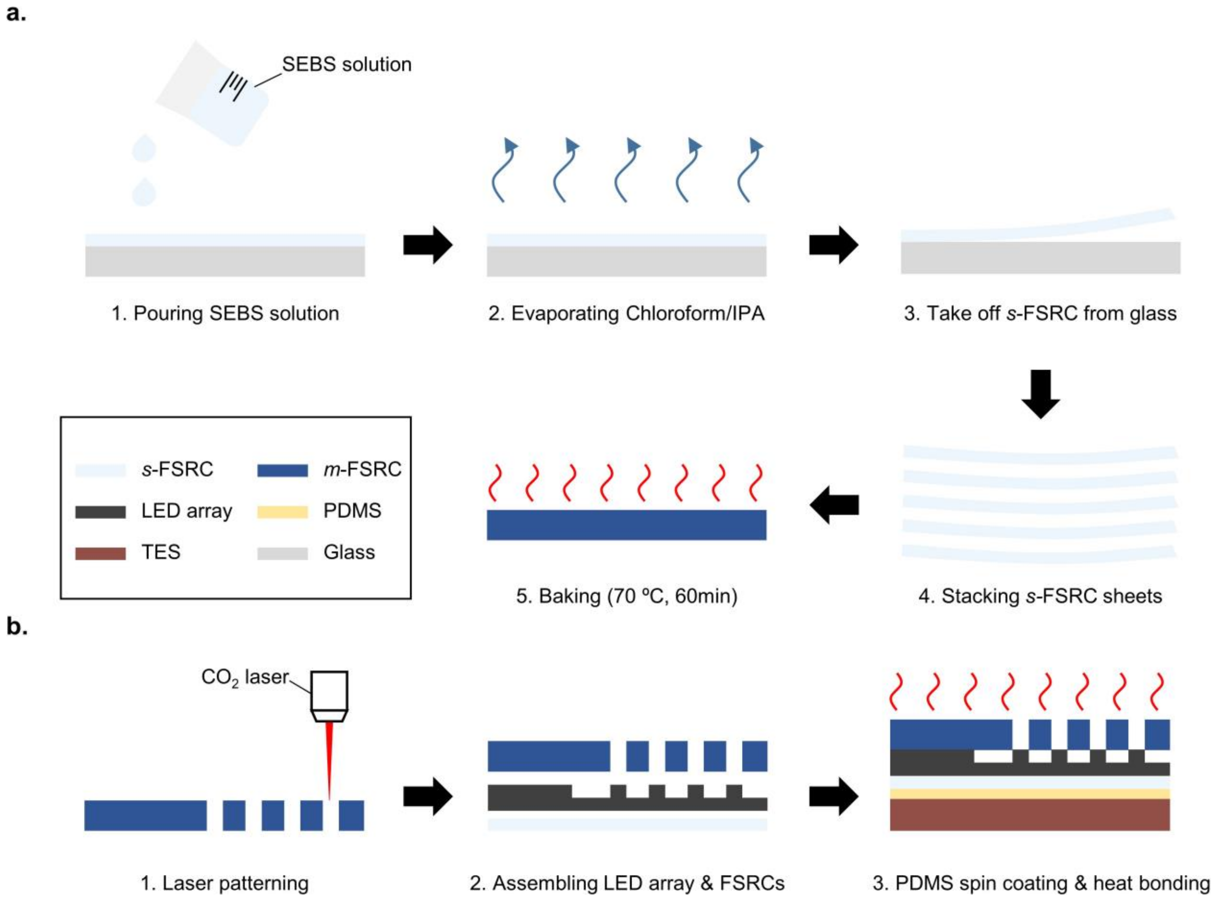

Figure S13. Fabrication process of a) *m*-FSRC and b) optoelectronic device with TES-RC design.

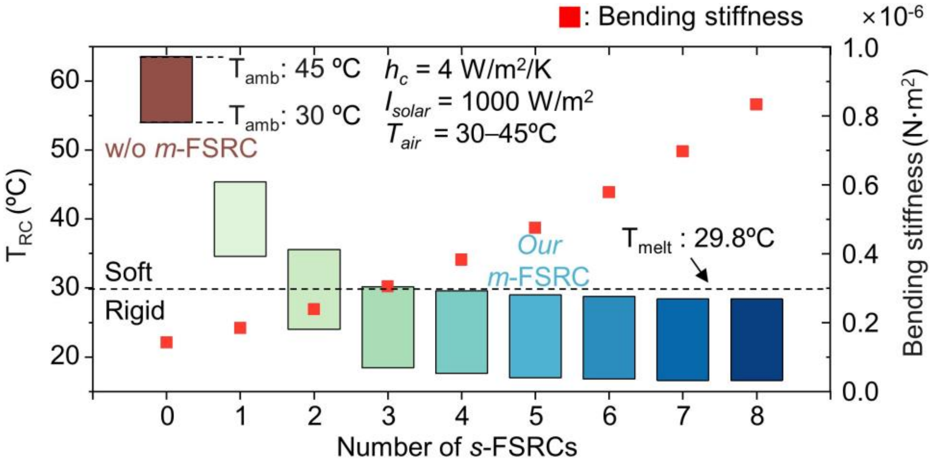

Figure S14. Cooling performance and bending stiffness of *m*-FSRC-integrated transformative platform according to the number of *s*-FSRCs to form *m*-FSRC.

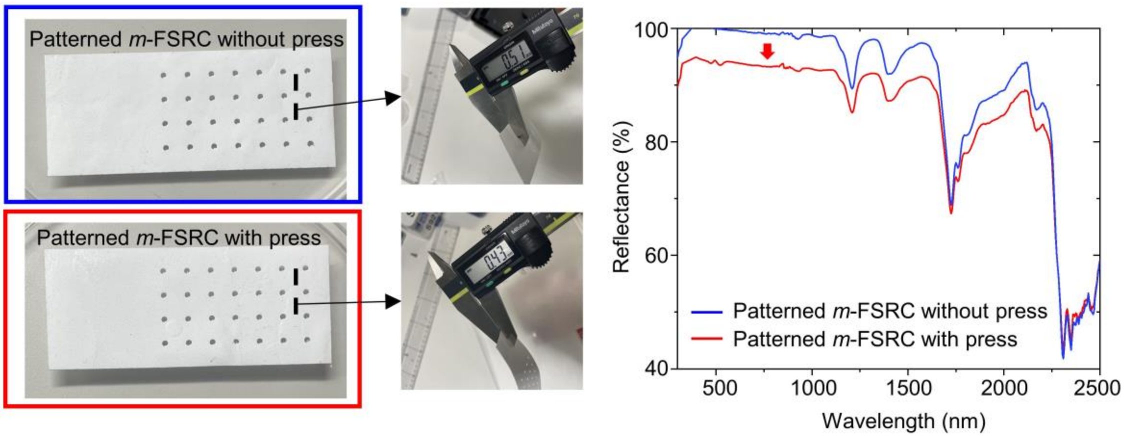

Figure S15. Comparison of *m*-FSRCs fabricated by thermal bonding without press and with press.
